# Development of a behaviour change intervention: a case study on the practical application of theory

**DOI:** 10.1186/1748-5908-9-42

**Published:** 2014-04-03

**Authors:** Mark Porcheret, Chris Main, Peter Croft, Robert McKinley, Andrew Hassell, Krysia Dziedzic

**Affiliations:** 1 Research Institute for Primary Care and Health Sciences, Keele University, Keele, Staffordshire ST5 5BG, UK; 2 Keele University Medical School, Keele University, Keele, Staffordshire ST5 5BG, UK

**Keywords:** Behaviour change intervention, Theory, General practice, Osteoarthritis, Complex intervention, Implementation, Consultation, General practitioners, Communication skills

## Abstract

**Background:**

Use of theory in implementation of complex interventions is widely recommended. A complex trial intervention, to enhance self-management support for people with osteoarthritis (OA) in primary care, needed to be implemented in the Managing Osteoarthritis in Consultations (MOSAICS) trial. One component of the trial intervention was delivery by general practitioners (GPs) of an enhanced consultation for patients with OA. The aim of our case study is to describe the systematic selection and use of theory to develop a behaviour change intervention to implement GP delivery of the enhanced consultation.

**Methods:**

The development of the behaviour change intervention was guided by four theoretical models/frameworks: i) an implementation of change model to guide overall approach, ii) the Theoretical Domains Framework (TDF) to identify relevant determinants of change, iii) a model for the selection of behaviour change techniques to address identified determinants of behaviour change, and iv) the principles of adult learning. Methods and measures to evaluate impact of the behaviour change intervention were identified.

**Results:**

The behaviour change intervention presented the GPs with a well-defined proposal for change; addressed seven of the TDF domains (*e.g*., knowledge, skills, motivation and goals); incorporated ten behaviour change techniques (*e.g*., information provision, skills rehearsal, persuasive communication); and was delivered in workshops that valued the expertise and professional values of GPs. The workshops used a mixture of interactive and didactic sessions, were facilitated by opinion leaders, and utilised ‘context-bound communication skills training.’ Methods and measures selected to evaluate the behaviour change intervention included: appraisal of satisfaction with workshops, GP report of intention to practise and an assessment of video-recorded consultations of GPs with patients with OA.

**Conclusions:**

A stepped approach to the development of a behaviour change intervention, with the utilisation of theoretical frameworks to identify determinants of change matched with behaviour change techniques, has enabled a systematic and theory-driven development of an intervention designed to enhance consultations by GPs for patients with OA. The success of the behaviour change intervention in practice will be evaluated in the context of the MOSAICS trial as a whole, and will inform understanding of practice level and patient outcomes in the trial.

## Background

Osteoarthritis (OA) is a highly prevalent condition in general practice, and guidance on its management is available [1-6]. Published surveys of current practice have identified that care is not being delivered as recommended in this guidance, indicating that there is a need to improve and optimise primary care of people with OA [7-9].

**Figure 1:**
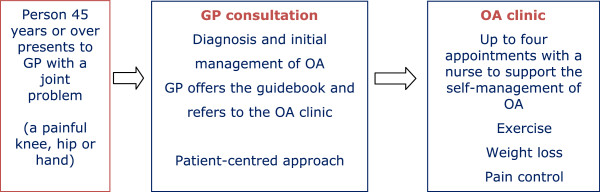
The MOSAICS trial intervention for enhancing osteoarthritis (OA) care.

The case study described in this paper was a component of the Managing Osteoarthritis in Consultations (MOSAICS) trial [10], an investigation of the feasibility, acceptability and impact of implementing the National Institute for Health and Care Excellence (NICE) OA Guideline [2]. The main aim of the MOSAICS study was to test a complex patient-focused intervention (the ‘trial intervention’), developed using the Whole Systems Informing Self-Management Engagement (WISE) model [11] and incorporating the three elements of that model: information for patients, professional responsiveness to patients’ needs, and access to care. The three elements in the trial intervention were: i) an OA Guidebook developed with user involvement to provide patient-centred and evidence-based information [12], ii) an enhanced OA consultation by GPs and practice nurses, and iii) access to a practice-based nurse-led OA clinic (providing an initial 30-minute appointment and up to three further 20-minute appointments to provide support for self-management). The intervention was an evidence-based service for people who were 45 years or older presenting to the practice with a peripheral joint problem (Figure 1), designed to provide: i) relevant written information for patients, ii) support for patients to undertake muscle strengthening exercises, increase physical activity and, if applicable, lose weight, and iii) advice to patients on the appropriate use of analgesia. Its impact is to be evaluated at the level of the practice, for example prescribing patterns and the recording of clinical information, and at the level of the patient, for example uptake of NICE recommended treatments and pain.

The Medical Research Council’s (MRC) updated guidance on the development and evaluation of complex interventions highlights the need to ensure successful implementation of interventions in research settings, and that failure to do this can undermine the evaluation of the intervention being tested [13]. This often requires a change in clinical practice by those delivering the intervention, and there is a growing evidence base on developing, undertaking and evaluating interventions to effect specific changes in professional behaviour: behaviour change interventions [14]. One component of implementing the MOSAICS trial intervention was to enhance the consultation behaviour of the GPs delivering the trial intervention. This behaviour concerned diagnosis and initial management in line with the NICE OA Guideline when patients aged 45 years and over present with peripheral joint pain. This GP behaviour was the focus of the case study described here.

The use of theory to inform the development of behaviour change interventions is strongly advocated by experts in the field [15-17] and is often presented as a model or framework. In this paper, we use ‘model’ as shorthand for a theoretically derived model or framework. Our case study comprises a description of the systematic selection and use of models to inform development of a behaviour change intervention designed to change GP clinical practice during consultations with patients with OA.

## Methods

**Figure 2:**
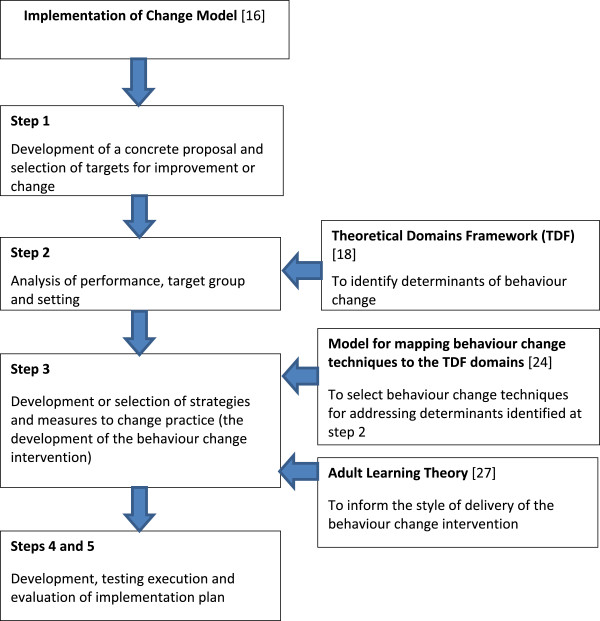
Models used for the development and delivery of the behaviour change intervention.

Four models were selected for their ability to operationalize the aims of the MOSAICS study in relation to the behaviour desired of GPs in the study, and their order of use is shown in Figure 2.

### The implementation of change model

This model, developed by Grol and Wensing [16], was selected to inform the overall approach to developing the behaviour change intervention. It comprises five steps: first developing a ‘concrete proposal’ for the desired change, one that is clearly defined and easily understandable; second undertaking an analysis of current practice, and barriers and incentives for change, in the group in which change is desired; third developing and selecting ways to change practice; and finally (steps 4 and 5) undertaking and evaluating the implementation plan (Table 1). Detailed guidance is available on how to approach the tasks needed for each step with reference to the underpinning evidence [16], and was selected as, in addition to its logical approach, it provides guidance on the answers to three very practical questions during the planning of change: ‘where do we want to be?’ (step 1), ‘where are we now?’ (step 2), and ‘how do we get there?’ (step 3).

**Table 1:** **Implementation of change model – adapted from Grol *et al*. [16]**

**Step**	**Summary of activities**
1	Development of a concrete proposal and targets for improvement or change
	• Systematic development
	• Involvement of target group
	• Good ‘product’
	• Accessible and attractive form
	• Opportunity for local adaptions
2	Analysis of performance, target group and setting
	• Stakeholders
	• Current practice
	• Barriers and incentives
	• Readiness to change of subgroups
3	Development or selection of strategies and measures to change practice
	• Tailored to target group and/or setting
	• Cost-effective mixture of techniques of proven value
	• Strategies for implementation
4	Development, testing and execution of implementation plan
5	Evaluate and, where necessary, adapt plan

### The theoretical domains framework

At step 2, a key task was to understand which factors, or ‘determinants,’ would impede or facilitate the intended change, and many psychologically-oriented models have been proposed to inform this task. Many of these models overlap, and each tends to focus on different aspects of the change process [16]. One challenge for those facilitating change is how to select the most appropriate model when undertaking an analysis of these factors in a particular set of circumstances. Michie *et al.* addressed this problem by undertaking a consensus exercise to develop a model that encompassed 128 theoretical constructs (or determinants) included in 33 psychological theories - the Theoretical Domains Framework (TDF) [18]. The TDF consists of 12 domains (Table 2), such as knowledge, skills, beliefs about consequences, motivation and goals, with each domain having a set of theoretical constructs that had been identified as components in the models included in the consensus exercise. A total of 11 out of the 12 domains concern characteristics of the people for whom change is desired, with the 12^th^ concerning the attributes of the change or desired behaviour itself. The TDF has been used to identify determinants of behaviour change for an extensive range of conditions and clinical situations, for example, mobilisation of older patients in hospital [19], utilisation of a rule for the use of CT scans for head trauma [20], and management of chronic obstructive airways disease [21], and its development and use in a range of other studies has been reviewed [22]. The TDF has been recently validated and refined: experts were asked to re-sort the constructs included in the TDF and to re-develop the domains, with and without reference to the original domains [23]. The refined framework consists of 14 domains, 8 unchanged from the original, 6 derived from a more specific grouping of the constructs underpinning 3 of the domains (beliefs about capabilities, beliefs about consequences, and motivation and goals), with 1 of the original domains omitted (nature of the behaviour). The 12-domain TDF model was selected as the domains in this framework provided a practical and comprehensive list of possible determinants of behaviour change (the 14-domain model had yet to be developed at the time of this study), and the TDF was utilised to identify relevant determinants of behaviour change in this study.

**Table 2:** **Theoretical Domains Framework adapted from Michie *et al*. [18]**

**TDF Domain**	**Example of use of domain when assessing target group concerning a behaviour change ‘X’**
Knowledge	Are they aware of X?
Skills	Do they know how to do X?
Social/professional role and identity	Is X compatible with professional identity?
Beliefs about capabilities	How confident are they that they can do X?
Beliefs about consequences	What do they think will happen if they do X?
Motivation and goals	How much do they want to do X?
Memory, attention and decision processes	Will they remember to do X?
Environmental context and resources	Are there physical or resource factors which will facilitate or hinder X?
Social influences	Will they observe others doing X?
Emotion	Does X evoke an emotional response?
Behavioural regulation	What preparatory steps are needed to do X?
Nature of the behaviour	How understandable is X?

### Model for mapping behaviour change techniques to the TDF domains

At step 3, one of our tasks was to develop or select techniques to effect behaviour change. Michie *et al.* developed a model to inform the selection of behaviour change techniques that target the determinants described in the TDF [24]. They identified, and defined, a set of behaviour change techniques described in the literature and mapped them to the domains in the TDF described above (barring the 12^th^ domain): the techniques that they judged to be effective in changing behaviour for each domain [24]. The approach to mapping behaviour change techniques to TDF domains has been incorporated into protocols for the development of complex interventions, for example for tobacco counselling in dentistry [25] and management of low back pain [26]. This mapping process provides a practical tool for selecting appropriate behaviour change techniques as the components of a behaviour change intervention and was utilised at step 3.

### Adult learning theory

At step 3, the principles of adult learning theory were also utilised; that adults are internally motivated and self-directed, bring life experiences and knowledge to learning experiences, are goal and relevancy oriented, are practical and like to be respected [27]. Adult learning theory was selected to inform the educational process of the behaviour change intervention as it has a well-established role in development of courses to support continuing professional development [27], including interventions such as the one developed in this study.

### Applying the models

#### Step 1 – development of a concrete proposal for change

The behaviour change required of the GPs was the delivery of an enhanced OA consultation (see Figure 1). A consensus exercise was undertaken with healthcare professionals to develop a model for the OA consultation [28]. Subsequent to this, two activities were undertaken. Firstly, the characteristics of the consensus model OA consultation were compared with characteristics known to promote or hinder the implementation of an innovation [16]. Secondly, three general practice advisory groups were formed – two consisting of GPs with research or teaching roles at Keele University and one consisting of members of the primary healthcare team in a local general practice – and meetings arranged. The meetings were audiotaped and field notes made. The model OA consultation was presented to the groups and their views and understanding obtained. From the results of the comparison and feedback from the advisory groups, the model consultation was refined to enhance uptake by GPs.

#### Step 2 – analysis of performance, target group and setting

The advisory groups, at the same meetings as arranged for step 1, were asked about: i) their current management of OA, ii) their awareness of, and agreement with, the NICE OA Guideline, and iii) any gaps perceived between their current practice and that recommended by NICE and in the model consultation. In addition, they were asked to suggest which barriers and/or incentives might be relevant to implementing the model consultation in practice. Their responses were mapped by the study team to the domains in the TDF.

#### Step 3 – development or selection of strategies and measures to change practice

There were four phases to the development of the behaviour change intervention: defining content, selecting behaviour change techniques, deciding on style of delivery, and addressing local practicalities. The content was developed by the study team informed by the views of GPs from step 2. The mapping of behaviour change techniques to TDF domains was utilised to select the techniques to address domains identified in step 2. Adult learning principles and Cochrane Effective Practice and Organisation of Care Group’s reviews [29] were used to decide on style of delivery. Practical issues, such as venues, timings and duration of meetings, how best to deliver the behaviour change intervention, and what was feasible in the MOSAICS study, were addressed by the study team in consultation with general practices in the study.

#### Steps 4 and 5 – development, testing and execution of the implementation plan, and its evaluation

The GP behaviour change intervention was undertaken as part of the MOSAICS study in practices randomised to the intervention arm of the study. Methods and measures were developed to evaluate the behaviour change intervention at five levels: satisfaction with delivery of the behaviour change intervention, mediators of change, self-reported intended behaviour, competency to undertake the behaviour (undertaking the behaviour in a controlled situation [30]), and performance in undertaking the behaviour in day-to-day practice.

## Results

### Step 1 – development of a concrete proposal for change

The model OA consultation, developed by the consensus exercise, consisted of 25 tasks addressing: i) assessment of chronic joint pain, ii) patient’s ideas and concerns, iii) exclusion of red flags, iv) examination, v) provision of the diagnosis and written information, vi) promotion of exercise and weight loss, vii) initial pain management, and viii) arrangement of a follow-up appointment [28].

The advisory group meetings were led by one of the authors (MP) and attended by 15 GPs, 5 practice nurses, and a practice manager. The key finding from the meetings on the characteristics of the model OA consultation was that, presented as 25 tasks, it was too complex to explain simply and quickly to GPs or for them to easily understand and translate into day-to-day practice. To simplify the model, tasks were grouped by core elements of a patient-centred consultation [11,31,32], for example support for self-care and provision of evidence-based information, and the model succinctly presented as three tasks.

1. To make, give and explain the diagnosis.

2. To provide analgesia advice/prescription.

3. To promote and support self-management.

### Step 2 – analysis of performance, target group and setting

The advisory group meeting transcripts and field notes on current practice, attitudes to recommended best practice, and perceived barriers to, and incentives for, changing practice, were analysed using the TDF as a coding framework. The analysis was discussed by the study team and by a group of expert educational advisors to the study, and seven TDF domains were identified as relevant to changing GP practice in OA consultations (Table 3).

**Table 3:** Determinants for implementing the enhanced OA consultation ordered by Theoretical Domains Framework (TDF) domain

**TDF domain**	**Aspects of domain identified in target group analysis**
Knowledge	The epidemiology and impact of OA, the recommendations of the NICE OA Guideline, the rationale for GPs providing support for the self-management of OA and that of making the diagnosis of OA clinically, details of the MOSAIC study procedures
Skills	The skills needed to make the diagnosis of OA clinically, and those for delivering the model OA consultation
Social/professional role and identity	The credibility of NICE guidance in general and specifically of NICE OA guidance, and the GP’s role in providing support for self-management
Beliefs about capabilities	The time to deliver the model OA consultation in day-to-day practice, and any previous difficulties in managing OA
Beliefs about consequences	The GPs’ doubts about the efficacy of OA interventions recommended by NICE OA guidance
Motivation and goals	That OA and its management was not considered a high priority by the GPs, compared with other areas of general practice
Memory, attention and decision processes	The GPs remembering to undertake the model OA consultation in day-to-day practice, when an older adult presents with peripheral joint pain

### Step 3 – development or selection of strategies and measures to change practice

The content of the behaviour change intervention was derived by the study team from the practical requirements of delivering the model OA consultation and from gaps identified in the advisory group meetings, for example lack of knowledge about the impact of OA on the individual, the skills necessary to deliver the model OA consultation, and the credibility of NICE guidelines. The selection of behaviour change techniques was undertaken by the study team and the educational advisors to the study. The starting point was the list of techniques that Michie *et al*. had judged appropriate to effect change for domains identified in step 2 [24]. The group used their research, clinical and educational experience to decide which of these techniques to choose. The content of, and techniques to address, each domain are detailed in Table 4.

**Table 4:** Content of behaviour change intervention and behaviour change techniques by relevant domains of the Theoretical Domains Framework (TDF)

**TDF domain**	**Behaviour change intervention content**	**Techniques for behaviour change chosen to address domain**
Knowledge	Burden/prognosis/pathophysiology of OA, experience of patients with OA of general practice	Information provision to address gaps in knowledge about:
		● The nature and management of OA
		● NICE OA recommendations
	NICE OA guidance, efficacy OA treatments	● The model OA consultation
	Rationale for making the diagnosis of OA clinically and for giving the diagnosis	
	Rationale for self-care of OA, support for self-care and patient centre consulting	
	OA Guidebook and the model OA consultation	
Skills	Assessing ideas/concerns and expectations/treatment preferences	Rehearsal of relevant skills; graded task starting with easy tasks; increasing skills (problem-solving) to:
	Making a clinical diagnosis of OA	● Enhance GP consultation skills for OA
	Giving the diagnosis/explaining OA and its treatment (use of language)	
	Use of NICE recommended treatments	
	Promoting OA Guidebook and nurse follow-up appointment	
Social/professional role and identity	Attitudes to guidelines and NICE OA guidance	Social process of encouragement, pressure and support to:
	Attitudes to support for self-care (potential conflict between professional care and self-care)	● Engender a positive approach to guideline implementation and support for self-care
Beliefs about capabilities	Time to do it	Social processes of encouragement, pressure, support to:
	Other priorities in consultation	● Enhance perceived ability to deliver the model OA consultation
	Discussion about problems with managing OA/what would help to better manage it	
Beliefs about consequences	Discussion on beliefs about consequences of OA interventions and model OA consultation	Information provision; persuasive communication to:
		● Counter perceived lack of efficacy of interventions for OA
Motivation and goals	Presentation of MOSAIC study payments	Contract; rewards; persuasive communication to:
	Provision of practice nurse training and a lifestyle change intervention	● Sign GPs up to delivering the model OA consultation
Memory attention and decision processes	Model OA Consultation Aide Memoire	Prompts, triggers, cues to:
		● Prompt delivery if model OA consultation in day-to-day practice

The choice of delivery style was informed by evidence from the Cochrane Effective Practice and Organisation of Care Group on the effectiveness of strategies for changing practice, with a specific emphasis on small group learning with a mixture of didactic and interactive sessions [33] and facilitated by opinion leaders [34]. In addition, the study team drew on evidence on a learner-centred approach, which utilises prior knowledge and experiences of the participants [27] to effect change in behaviour. Specifically, for the delivery of techniques to address the skills domain, we used empirical evidence on techniques for training experienced GPs in communication skills, a method of training known as ‘context-bound communication skills training’ was adopted [35]. In this technique the ‘context,’ in this case the management of OA, is in the foreground and the communication training in the background. A key feature is that participants practise consultation skills when consulting with simulated patients and receive feedback. This had been found to be a feasible, acceptable and effective method of enhancing the consultation skills of experienced practitioners [36] and preferable, for this group, to the approach taken in undergraduate skills teaching, where it is skill and not context that is in the foreground.

The final step was to consider the practical issues in delivering the workshops in four general practices with all the myriad demands on the GPs’ and other practice staff’s time. The final format was developed by the study team and educational advisors, drawing on their professional experience, and in consultation with GPs working in Keele University Medical School. The format was to deliver the behaviour change intervention at general practices’ premises, in four sessions, lasting one or two hours each, and about two to three weeks apart. The final behaviour change intervention with detailed timings is shown in Table 5.

**Table 5:** Workshop schedules to deliver the behaviour change intervention for GPs in the MOSAICS trial

**Workshop 1 – attendees: Primary Health Care Team from a single practice (GPs, practice nurses, practice manager**^**1**^**, receptionists**^**1**^**) Duration: 2 hours**	
**Time (minutes)**	**Activity**
5	Introductions – facilitators and practice attendees.
20	How is OA managed, in your practice? Mapping practice, and local community and secondary care, resources for OA (interactive session with discussion recorded on flip chart).
25	OA knowledge update on: pathophysiology, definition and diagnosis, prevalence, prognosis and patient experience of OA (didactic session with discussion).
10	Information on: the NICE OA Guideline, support for self-management, the OA Guidebook, the model OA consultation (didactic session with discussion).
5	Break and non-clinical staff leave.
20	Presentation and discussion of case histories (GPs previously requested to bring). Difficulties in managing OA - what do GPs and nurses want from the sessions and what would aid them in managing OA (interactive session with issues recorded on flipchart and to be addressed in workshop 3).
25	Details of the model OA consultation - how to deliver it in day-to-day practice - GP and practice nurse roles. Aide-memoire introduced (didactic session with discussion).
10	Conclusion and outline of workshops 2 and 3. GPs givenDVD of simulated patient consultation^2^ and asked to view in preparation for workshop 2.
**Workshop 2 – Attendees: GPs from two practices.**^**3**^**Duration: 2 hours**	
10	Introductions – facilitators and GPs. Reflection on, and unanswered questions from, workshop 1.
20	Discussion and reflection on video-recorded simulated patient OA consultations. Comparison between current practice and model OA consultation. Agenda for skills training agreed (interactive session with “agenda” recorded on flipchart).
10	Introduction to skills training: description of purpose and methods - the GPs were asked to work as a team trying out in turn bite-sized parts of the consultation with discussion and feedback from colleagues and facilitators (didactic session with discussion).
10	Break.
60	Skills training: working through the agenda set earlier. Particular emphasis on communication, use of language for giving and explaining the diagnosis and patient-centred approach (led by an experienced GP educator).
10	Reflection and conclusion. Aide-memoire discussed. Preparation for second video-recorded simulated patient consultation.^4^ Outline of workshop 3.
**Workshop 3 – Attendees: GPs from two practices. Duration: 2 hours**	
40	Knowledge update: addressing needs identified in workshop 1 and questions from GPs, and covering: diagnosing OA clinically and ‘top tips’ for managing OA (interactive session led by academic rheumatologist).
10	Discussion and reflection on 2nd video-recorded consultation. Agenda for skills training agreed (interactive session with “agenda” recorded on flipchart).
10	Break.
50	Skills training: as for workshop 2.
10	Conclusion and general reflection. Aide-memoire discussed. GPs invited to complete satisfaction questionnaires. Outline of workshop 4.
**Workshop 4 – Attendees: GPs and practice nurses from a single practice. Duration: 1 hour**	
40	Action planning on delivery of the model OA consultation in the practice. Final version of the aide-memoire agreed.
10	Presentation of baseline data on OA consultations in the practice (an OA data collection template had been installed in the practices for the six months prior to the training).
10	Conclusion and thanks. Attendance certificates issued.

1 - For first hour only.

2 - All GPs were invited to undertake a video-recorded consultation with a simulated OA patient prior to workshop 1.

3 - GPs from two practices came together for workshops 2 and 3.

4 - All GPs were invited to undertake a 2nd video-recorded consultation between workshops 2 and 3.

### Steps 4 and 5 – development, testing and execution of the implementation plan, and its evaluation

All the GPs, practices nurses, and administrative staff working in the four practices randomised to the intervention arm of the MOSAICS study, were invited to attend the training sessions (see Table 5 for details) [10]. The GPs were invited to participate in the evaluation of the behaviour change intervention. Methods and measures were chosen and developed to evaluate the behaviour change intervention at the four levels (Table 6).

**Table 6:** Methods and measures to evaluate the behaviour change intervention

**Evaluation level**	**Method**	**Measure**
Satisfaction with workshops (delivery of behaviour change intervention).	Questionnaire administered at the end of workshop 3.	Level 1 Kirkpatrick educational outcomes [37], such as level of enjoyment, views on content and confidence in delivering the model OA consultation.
Intention to practise.	Questionnaire administered before and twice after (at one month and five months after) the behaviour change intervention.	Vignette of an older adult presenting with joint pain and options for assessment and management.
Mediators of change.	Questionnaire administered before and twice after (at one month and five months after) the behaviour change intervention.	Statements based on TDF* domains identified at step 2, for example “How much do you think exercise and increasing physical activity by people with osteoarthritis will improve their pain (beliefs about consequences).
Competency in delivering the model OA consultation.	Video-recordings of the GPs undertaking a consultation with simulated OA patients were made before and twice after (at one and five months after) the behaviour change intervention.	Videos were assessed for the presence of specific behaviours necessary for the delivery of the model OA consultation.
Performance in delivering the model OA consultation.	Patient report: patients who attended the MOSAICS study nurse-led OA clinic were asked to report on the content of the previous GP consultation.	Four aspects of the consultation, did the GP: elicit ideas about the problem, give the diagnosis, explain the diagnosis, hand out the guidebook?

*Theoretical Domains Framework.

## Discussion

The utilisation of the Grol and Wensing Implementation of Change Model, the Theoretical Domains Framework, and the model for mapping behaviour change techniques to the TDF domains have enabled a systematic and theory-driven approach to be taken to the development of an intervention to change clinical practice for the management of OA by GPs, and measures to evaluate its impact. This proved to be a practical way of using theory to inform, rather than just inspire, the development of a complex intervention, an approach that is widely advocated but reportedly not always taken [15,38-40].

The Grol and Wensing model did enable us to answer the three questions ‘where do we want to be?’, ‘where are we now?’, and ‘how do we get there?’ – a task that is recommended in the MRC guidance on complex interventions: that researchers can fully describe important components of the overall intervention and can implement them in the research setting [13]. The use of the TDF at step 2, and behaviour change technique mapping at step 3, enabled identification of relevant determinants of change in the GP behaviour component of the main trial, and behaviour change techniques to address them, within specific theoretical frameworks. It also enabled the purpose of each item of the behaviour change intervention to be understood, for example information giving to address gaps in knowledge about OA, rehearsal and feedback to enhance consultation skills.

In addition to theory, empirical evidence and practical considerations, on style and mode of delivery, informed development and ensured that the end product was evidence-based, feasible to deliver and acceptable to the recipients.

### Use of models to develop behaviour change interventions in other studies

The TDF and behaviour change technique mapping, developed by Michie *et al*., have both been published within the last 10 years, and a number of studies have reported on utility and outcome in the development of behaviour change interventions for trials [26,41,42]. Both models, used sequentially as in this study, have been employed in development of interventions to improve management of low back pain [26], to enhance GP diagnosis of dementia [41], and to reduce antibiotic use for upper respiratory infections [42]. Two of these have resulted in multi-facetted interventions as developed in this study [26,41], with the other [42] resulting in two interventions, each specifically addressing one of two determinants of behaviour change identified. The research team in the low back pain study, having determined the behaviour change techniques to include in the intervention, and the mode of delivery, took a pragmatic approach to their final selection: what was locally feasible and acceptable. We also took a pragmatic approach on deciding the final format, but this did not result in any changes to our intended delivery other than that the workshops were run at the practices, lasted no more than two hours each, and were about two to three weeks apart. To date, only the low back pain trial has reported and showed a small effect on GP intention to practice but no significant change in actual behaviour [43]. That clinical practice was not observed to change may not have been due to the intervention per se, as there were logistical problems in getting GPs to attend the intervention workshops and methodological problems in assessing outcome. The drive to use theory to inform development of interventions has been questioned [44], as empirical evidence is lacking on effectiveness of interventions developed in this way. Although the low back pain trial did not demonstrate a change in clinical practice, its use of theory does add to empirical evidence on the process of behaviour change.

### Strengths and possible limitations

Developing complex interventions is a complex task in itself, and understanding how to approach it in a systematic way, informed by relevant theory, can be daunting for research teams [13]. The principal strength of the method described in this paper is that it enabled the MRC guidance on developing complex interventions to be operationalized systematically, and in a practical and do-able manner. The guidance on using the Grol and Wensing model to change clinical behaviour is extensive [16] and provided a very usable manual on ‘how to do it.’ The use of the TDF strengthens the approach advocated for the Grol and Wensing model for step 2, and is reflected in the increasing popularity of the TDF by research teams in developing interventions [22]. In addition, the recent validation and refining of the TDF domains has strengthened the rationale for its methodology, as used in this study, and, with a refined structure, strengthened its use in future studies [23].

The use of GP advisory group meetings both to gain views about the proposed change (step 1) and to undertake the target group analysis (step 2) was a practical strength. It provided an efficient method of: i) involving the target group in the development of the change proposal (an activity it its own right that enhances uptake of an intervention [16]), ii) identifying which characteristics of the intervention might hinder or facilitate uptake, and iii) understanding current practice and identifying relevant determinants of change.

One potential limitation was that the topic guide for the advisory group meetings was not specifically developed from the TDF, which could have resulted in some of the TDF domains not being fully explored in the meetings. The topic guide had been developed, and the meetings undertaken, before deciding to use the TDF in step 2. However, the topic guide was broad and covered current management, views about recommended practice, and perceived gaps between current and recommended care and allowed for free discussion by the groups. This has occurred in other studies [21,45] and, although not used to develop the topic guide, the TDF did give an efficient method for analysing advisory group comments.

The GPs who attended advisory group meetings were not the same GPs who received the behaviour change intervention in the MOSAICS trial, and their views and attitudes may not have been the same as these GPs. Analysis of the actual target group for the behaviour change intervention – the GPs in the four MOSAICS intervention practices – may have identified different determinants to be addressed, but the timescale for developing the behaviour change intervention in the MOSAICS study did not allow for this. However, as the mode of delivery included interactive sessions, and the sessions encouraged reflection on current practice and on the video-recorded consultations, there was ample opportunity for issues specific to the study GPs to be addressed.

The final measure of success, beyond the fact that this methodology has provided the framework for an intervention deliverable in practice, is whether it achieved what it set out to (a change in clinical practice) in a sufficient dose to achieve optimal outcomes for patients in the MOSAICS trial. Both these outcomes (intermediate professional-focused and ultimate patient-focused) will be reported in the future as part of the main results from the MOSAICS study.

## Conclusion

A stepped approach to the development of a professionally-focussed behaviour change intervention to implement a component of a trial intervention, with the utilisation of theoretical frameworks to identify determinants of change and match behaviour change techniques to these, has enabled the systematic and theory-driven development of an intervention to enhance the management of OA by GPs. The success of the behaviour change intervention will be evaluated in the context of the MOSAICS trial, and will inform the understanding of practice level and patient outcomes in the trial.

## Competing interests

None of the authors have any competing interests to declare.

## Authors’ contributions

MP developed the methodology, facilitated the advisory groups, undertook the data analysis and drafted the manuscript. CM and KD participated in developing the methodology, facilitating the advisory groups, analysing data, and drafting the manuscript. PC, RMcK and AH participated in developing the methodology, analysing data, and drafting the manuscript. All authors read and approved the final manuscript.
